# Permanent interstitial ^125^I seed implantation as a salvage therapy for pediatric recurrent or metastatic soft tissue sarcoma after multidisciplinary treatment

**DOI:** 10.1186/s12957-015-0747-7

**Published:** 2015-12-15

**Authors:** Lihong Yao, Junjie Wang, Yuliang Jiang, Jinna Li, Lei Lin, Weiqiang Ran, Chen Liu

**Affiliations:** Department of Radiation Oncology, Peking University Third Hospital, Hua-yuan North Road No.49, Beijing, 100191 People’s Republic of China; Department of Ultrasound, Peking University Third Hospital, Beijing, 100191 People’s Republic of China; Department of Radiology, Peking University Third Hospital, Beijing, 100191 People’s Republic of China

**Keywords:** ^125^I seed, Pediatric, Soft tissue sarcoma, Image guided

## Abstract

**Background:**

The management of pediatric recurrent or metastatic soft tissue sarcoma after multimodal treatment remains challenging. We investigated the feasibility, efficacy, and morbidity of permanent interstitial ^125^I seed implantation under image guidance as a salvage treatment for pediatric patients with recurrent or metastatic soft tissue sarcoma.

**Methods:**

This was a retrospective study of 10 patients who underwent percutaneous ultrasound or computed tomography (CT) guided permanent ^125^I seed implantation. Postoperative dosimetry was performed for all patients. Actuarial D90 was 121–187.1 Gy (median, 170.3 Gy). The number of ^125^I seeds implanted was 6–158 (median, 34.5), with a median specific activity of 0.7 mCi per seed (range, 0.62–0.8 mCi); total activity was 4.2–113.76 mCi. Follow-up time was 6–107 months (median, 27.5 months); no patients were lost to follow-up.

**Results:**

The overall response rate (complete response + partial response) was 8/10 (80 %), including two patients with complete response (CR) (20 %) and five patients with partial response (PR) (60 %). Local control rates after 1 and 2 years were 70.1 and 62.3 %, respectively, with a mean local control time of 70.6 months (95 % confidence interval (CI) 45.1–96.0). Survival rates after 1 and 2 years were 68.6 and 57.1 %, respectively, with a mean survival time of 65.3 months (95 % CI 34.1–96.5). Three patients died from distant metastasis; one died from local recurrence 12 months after seed implantation. Three patients suffered a grade I skin reaction and one developed ulceration. No severe adverse neurologic sequelae or blood vessel damage occurred.

**Conclusions:**

Image guided permanent interstitial ^125^I seed implantation as a salvage treatment appears to have a satisfactory outcome in children with recurrent or metastatic soft tissue sarcoma.

## Background

Soft tissue sarcomas (STSs) are rare mesenchymal tumors that represent 7–10 % of pediatric malignancies [1]. Although external beam radiotherapy (EBRT) after surgery has greatly improved local control, a series of clinical reports indicates that the locoregional recurrence rate is still about 8–20 % even after such management. Patients with local recurrence or metastasis of STS usually have a poor prognosis [1–4]. Recently, new therapeutic regimens for pediatric recurrent and metastatic STS have been developed; however, these have not resulted in favorable local control or survival rates, and the management of local recurrence and distant metastases thus remains challenging and nonstandardized. These difficulties are exacerbated by the fact that most pediatric patients undergo complex multidisciplinary management including numerous surgical procedures, radiotherapy, chemotherapy, and various combinations of these treatments.

In pediatric patients with recurrent or metastatic STS, local control may be regained by further surgery with adequate margins (wide or radical) and EBRT, though data on the survival benefit are limited. Adjuvant EBRT is known to be effective in reducing the recurrence rate [5] but is limited by the tolerance of the surrounding normal tissues or organs at risk (OARs), which makes it difficult to achieve a lethal dose to the sarcoma and ultimately leads to local recurrence or metastasis. Interstitial implantation of ^125^I seeds is a promising salvage therapy for many different recurrent and metastatic malignant tumors that has been used in, for example, re-recurrent rectal carcinoma [6], recurrent head and neck carcinoma [7], and metastatic lymph nodes [8, 9]. Interstitial permanent brachytherapy (BRT) brings new hope for the treatment of recurrent and metastatic STS in children because the radiation dose is well localized to the tumor bed and adjacent uninvolved tissues are spared [10–16]. However, the literature on experience with the use of permanent interstitial ^125^I seeds in pediatric patients is limited. It is therefore necessary to investigate the feasibility and efficacy of image guided permanent implantation of ^125^I seeds as a salvage therapy for locally recurrent STS in children and to determine local control, survival, and complications.

## Methods

### Patient information and selection

We retrospectively analyzed 10 pediatric patients (median age, 15 years; range, 4–20 years) with recurrence or metastasis of STS who underwent percutaneous ultrasound (color Doppler with probe and guidance stabilization devices; Aloka 550-5000) or computed tomography (CT) guided permanent ^125^I seed implantation at Peking University Third Hospital from December 2005 to March 2014. This study was approved by the ethics committee in Peking University Third Hospital and followed the guidelines for experimental investigation with human subjects required by our institution.

Eligibility criteria were as follows: written informed consent obtained from parents/guardians before seed implantation; the recurrent or metastatic tumor diagnosed by CT or magnetic resonance imaging (MRI); histologically proven recurrent STS after surgery; EBRT, chemotherapy, or a combination of these treatments; Karnofsky Performance Status score 60 or higher; and no severe dysfunction of the kidneys, liver, or bone marrow. Before implantation, the history of all patients was taken and physical examination, routine hematology and biochemistry, CT or ultrasonography of the lesions, and chest radiography were performed. Patients and primary tumor characteristics are shown in Table 1.

**Table 1:** Patient and primary tumor characteristics (*n* = 10)

	No. of patients	Percentage (%)
Age at implant (years)		
Median (range)	15 (4–20)	
Gender		
Male	7	70
Female	3	30
Pathology of primary tumor		
Fibrosarcoma	5	50
Alveolar soft tissue sarcoma	2	20
Primitive neuroectodermal tumor	1	10
Epithelioid sarcoma	1	10
Rhabdomyosarcoma	1	10
Primary tumor stage (AJCC 7th de, 2010)		
I A	3	30
II A	1	10
III	3	30
IV	3	30
Pre-seed implant therapy		
Surgery + EBRT + CTx	1	10
Surgery + EBRT	3	30
Surgery + CTx	1	10
EBRT + CTx	2	20
Surgery	3	30
Follow-up (month)		
Median (range)	27.5 (6–107)	

*Abbreviations*: *AJCC* American Joint Committee on Cancer, *EBRT* external beam radiotherapy, *CTx* chemotherapy

Seven of the 10 patients were boys, and three were girls. Two patients had re-recurrence after their third and fourth surgical procedures, respectively. One patient developed recurrence and metastasis after radical resection. Two patients who suffered recurrence after surgery received EBRT (median, 59 Gy; range, 58–60 Gy) but were re-recurrence. One patient experienced recurrence after surgery in combination with EBRT at 60 Gy. Two patients who were deemed unsuitable for surgery when initially reviewed suffered recurrence after adjuvant chemotherapy (median, 14 cycles; range, 12–16 cycles) and EBRT (median, 45.3 Gy; range, 33–57.5 Gy). One patient with pulmonary metastasis had recurrence disease after neoadjuvant chemotherapy (4 cycles), radical resection, EBRT (36 Gy), and adjuvant chemotherapy (8 cycles). In one patient, recurrence developed 17 months after initial intraoperative ^125^I seed implantation and subsequent chemotherapy (12 cycles). There were six cases of distant metastasis among the 10 patients; these caused mild to moderate pain and limited movement of the limbs to differing degrees. One patient developed blindness with exophthalmos due to compression of the right optic nerve by a metastasis. Two patients had difficulty opening their mouth. The treatment history before ^125^I seed implantation for each patient is listed in Table 2. All of the cases in this study had been interviewed by surgeons and radiation oncologists who had considered them unsuitable for salvage surgery and EBRT or the parents/guardians had refused to undergo surgery or EBRT.

**Table 2:** Treatment characteristics before ^125^I seed implantation (*n* = 10)

						Previous treatment				
No.	Gender	Age (years)	Stage^a^	Pathology	Position of implant seeds (recurrent position)	Surgery (times)	EBRT (courses)	Previous cumulative dose (Gy)	Chemotherapy (cycles)	Recurrent time^b^ (months)
1	F	16	T1aN0M0G1	Fibrosarcoma	Right axilla	1	1	60	0	12
					Right upper arm					
2	M	20	T1aN1M0G1	Fibrosarcoma	Left neck and left supraclavicular	2	1	60	0	24
					Before the left clavicle					
					left carotid artery					
3	F	19	T2bN0M1G2	Fibrosarcoma	Maxillofacial and right eye socket	1	0	0	0	17
4	M	4	T1bN0M0G2	Alveolar soft tissue sarcoma	Left tongue root	0	1	57.5	16	10
					Left tongue root and palatopharyngeal arch					
5	M	14	T1bN0M0G1	Fibrosarcoma	Right shoulder and neck	1	1	58	0	8
6	M	20	T1aN0M0G1	Fibrosarcoma	Right chest wall	4	0	0	0	72
7	M	9	T1bN0M1G3	Primitive neuroectodermal tumor	Left mandible	1	0	0	12	17
8	M	16	T2bN1M0G2	Epithelioid sarcoma	Right thigh	3	0	0	0	1
9	F	6	T2bN0M1G2	Rhabdomyosarcoma	Pelvic	1	1	36	12	1
10	M	11	T2bN1M0G2	Alveolar soft tissue sarcoma	Right face	0	1	33	12	4

^a^Tumor node metastasis (TNM) stage according to the American Joint Committee on Cancer Staging Manual (2010); ^b^Recurrent time is the interval between seed implantation and the last treatment

### Pretreatment planning

One to two weeks before seed implantation, a detailed CT/ultrasound aided tumor volume study was performed for all patients. We obtained CT transverse images of the targets at 5 mm intervals. The images were transferred to a three-dimensional radiation therapy planning system (3D-TPS; Beijing Astro Technology Co. Ltd, Beijing, China). An experienced radiation oncologist outlined the gross tumor volume (GTV) and the OARs on each transverse image. The planning target volume included the entire GTV with a 0.5–1-cm margin that was covered by the 90 % isodose curve. The D90 (prescribed dose delivered to 90 % of the target volume) of seed implant was calculated by the 3D-TPS. The total number and activity of ^125^I seeds to be implanted were determined according to our experience in previous studies [6–8].

### Image guided seed implant protocol

Under adequate local or general anesthesia, five patients underwent seed implant under CT guidance and the remainder underwent seed implant under ultrasound guidance. After the target volume had been determined, 18-gauge needles were implanted into the mass and spaced at a distance of 1.0 cm in a parallel array, extending at least 0.5–1.0 cm beyond the margins of the tumor. The direction of the needles was adjusted to avoid large blood vessels. The exact puncture process depended primarily on the operator’s experience. ^125^I seeds (Model 6711; t_1/2_, 59.4 days; energy, 27.4–31.4 keV; half-value layer of lead, 0.0025 cm; half-value layer of tissue, 2.0 cm; Beijing Atom and High Technique Industries Inc., Beijing, China) were implanted using a Mick applicator (Mick Radio-Nuclear Instruments Inc., Mount Vernon, NY, USA), with spaces between seeds (center to center) of approximately 1.0 cm. The needles were then removed.

### Postimplantation dosimetry

**Fig. 1:**
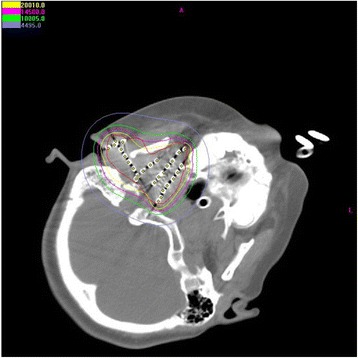
Isodose curve after seed implantation on a CT scan. The *inner red curve* represents the gross tumor volume. The ellipses are isodose lines of 200, 145, 100, and 45 Gy from inside to outside, respectively

**Fig. 2:**
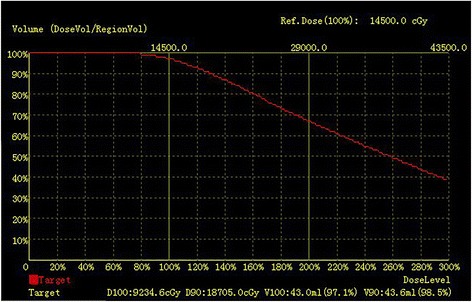
Dose–volume histogram for the target

Postoperative dosimetry was routinely performed for all patients immediately or 24 h after implantation using three-dimensional seed identification and 5-mm thickness CT scans. The contoured images and sources were entered into TPS software. Actual isodose distributions for each slice (Fig. 1) and dose–volume histograms for the target were generated (Fig. 2). Post-planning evaluation showed the actuarial D90 to be 121–187.1 Gy, with a median of 170.3 Gy. The number of ^125^I seeds implanted ranged from 6 to 158, with a median of 34.5. The median specific activity of the ^125^I seeds was 0.7 mCi per seed (range, 0.62–0.8 mCi). The total activity was 4.2–113.76 mCi.

### Definition of treatment response

Pain intensity was evaluated and graded according to the Numeric Rating Scale for chronic pain: 1–3 represented mild pain, 4–6 moderate pain, and 7–10 severe pain [17].

Local tumor response was evaluated by CT 2 months after seed implantation according to the Response Evaluation Criteria in Solid Tumors version 1.1 (RECIST) [18]. Briefly, complete response (CR) was defined as the complete disappearance of the lesion, without the appearance of any new lesions and then maintained for 4 weeks. Partial response (PR) referred to a more than 30 % decrease in the sum of the largest diameters of target lesions. Progressive disease (PD) was defined as at least a 20 % increase in the sum of the largest diameters of target lesions or the appearance of any new lesions. Stable disease (SD) was defined as neither sufficient shrinkage to qualify for PR nor sufficient increase to qualify for PD. The response rate was equal to the CR + PR.

Complications were scored using the Radiation Therapy Oncology Group (RTOG)/European Organisation for Research and Treatment of Cancer Late Radiation Morbidity Score Criteria [19].

### Follow-up

Patients were initially evaluated by radiation oncologists and surgeons 4 weeks after seed implantation and thereafter every 2–3 months, or more frequently if a new clinical sign or symptom appeared. After 2 years, the patients were followed-up every 6 months. Patient follow-up time was calculated from the date of seed implant and ranged from 7 to 107 months (median, 27.5 months); no patients were lost to follow-up. Disease status was assessed by physical examination, liver function tests, and complete blood and platelet counts. Imaging, including CT, MRI, and ultrasonography was used to confirm relapse events.

### Statistical analysis

Overall survival and local control rates were analyzed with PASW Statistics version 18.0 using the Kaplan–Meier method. Survival time was determined from the date of seed implantation to the date of death or the last follow-up. Deaths for any reason were scored as events when calculating survival rates. Local recurrence was defined as tumor progression within the implanted area or surrounding regions observed on CT, MRI, or ultrasonography.

## Results

### Response to treatment

Ten pediatric patients with 12 recurrent and two metastatic lesions were studied. After seed implant, the intensity of pain decreased to mild pain for the two patients who had suffered moderate pain and had difficulty opening their mouth. In the patient with exophthalmos, this was alleviated. One patient with limited right upper limb outreach recovered completely to normal. However, two other patients did not experience relief of limb movement restriction (one right upper limb, one right leg). ^125^I seed treatment details and outcomes are summarized in Table 3.

**Table 3:** Treatment characteristics of ^125^I seed implantation and outcomes (*n* = 10)

No.	Metastasis	Size (cm)	Type of seeds implant	Seed activity (mCi)	No. of seeds/D90 (Gy)	Post-seed implant treatment	RR	LR (m)	Overall survival (m)	Causes of death
1	Yes	4.0 × 2.9 × 2.2	CDU	0.7	33/164.5	No	CR	–	107	Survival
		1.3 × 1.5 × 1.0	CDU	0.7	6/148.1	No	CR	–		
2	Yes	4.0 × 3.6 × 1.8	CDU	0.8	50/172.7	Seed implant after progress	PR	24	70	Survival
		2.5 × 2.0 × 2.0	CDU	0.7	8/169.4	No	PR	–		
		1.8 × 1.2 × 1.0	CDU	0.65	9/171.6	No	PR	–		
3	Yes	6.6 × 7.8 × 9.5	CT	0.72	158/187.0	No	PR	12	14	LR
4	No	3.2 × 3.3 × 1.5	CDU	0.7	30/176.0	No	CR	–	62	Survival
		3.4 × 3.0 × 1.2	CDU	0.77	35/166.1	No	CR	–		
5	No	3.2 × 2.9 × 2.0	CDU	0.8	54/174.9	No	PR	–	51	Survival
6	No	4.0 × 2.0 × 3.0	CDU	0.62	40/121.0	No	PR	–	41	Survival
7	Yes	3.8 × 3.5 × 3.0	CT	0.72	34/167.2	EBRT (50Gy)	SD	8	12	MM
8	Yes	3.5 × 4.9 × 12	CT	0.72	157/171.2	No	PR	3	6	MM
9	Yes	3.6 × 2.0 × 3.0	CT	0.66	27/132.0	No	PD	3	6	MM
10	No	5.8 × 2.8 × 5.0	CT	0.68	86/187.1	No	PR	–	7	Survival

*Abbreviations*: *CDU* color Doppler ultrasound guided, *CT* computed tomography guided, *EBRT*, external beam radiotherapy, *RR* response rate, *CR* complete response, *PR* partial response, *SD* stable disease, *PD* progressive disease, *m* months, *LR* local recurrence, *MM* multiple metastasis

### Tumor local control

**Fig. 3:**
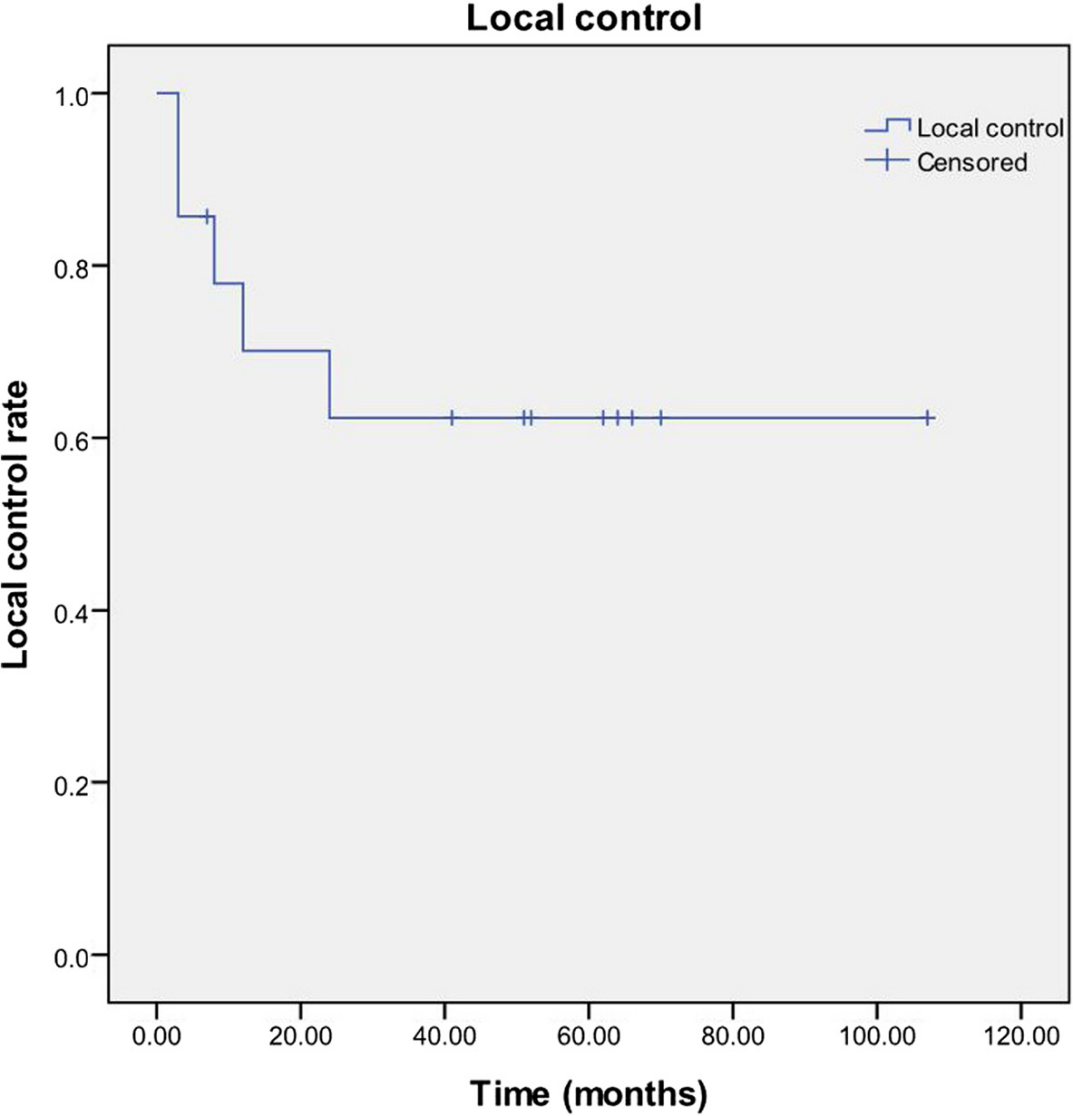
Actuarial local control curve for the 10 patients

The overall response rate (CR + PR) was 8/10 (80 %), including two patients with CR (20 %) and five patients with PR (60 %). One of the 10 patients had stable disease (SD; 10 %) and one had progressive disease (PD;10 %). Local control rates after 1 and 2 years were 70.1 and 62.3 %, respectively, with a mean local control time of 70.6 months (95 % confidence interval (CI) 45.1–96.0) (Fig. 3).

### Overall survival

**Fig. 4:**
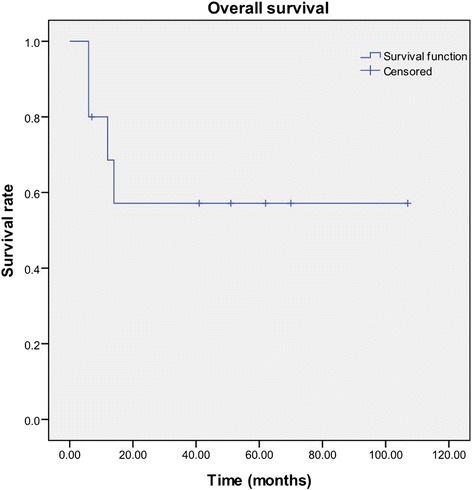
Actuarial survival curve for the 10 patients

Survival rates after 1 and 2 years were 68.6 and 57.1 %, respectively, with a mean survival time of 65.3 months (95 % CI 34.1–96.5) (Fig. 4). At the time of writing, three of the patients had died from multiple metastasis; one patient died from local recurrence 12 months after seed implantation. Six patients were still alive with no evidence of local recurrence or distant metastases.

### Toxicity and complications

Three patients suffered a grade I skin reaction that presented with local pigmentation after seed implantation. One patient developed an ulceration associated with disease progression and died due to pulmonary metastasis 6 months after seed implantation. No seeds were found to have migrated. No adverse neurologic sequelae or blood vessel damage occurred. No serious RTOG grade IV late complications were observed.

## Discussion

EBRT plays an important role in the treatment of STS in children. Radiotherapeutic approaches include EBRT, BRT, and intraoperative radiotherapy. However, EBRT can cause severe complications in the pediatric population, including growth retardation and effects on organ function. Although there are no randomized clinical trials comparing the curative effect of EBRT and BRT in patients with recurrent STS, theoretically, compared with EBRT, BRT has several advantages for pediatric patients with recurrent and/or metastatic STS after surgical resection. Due to its radiobiologic characteristics, BRT can deliver higher doses of radiation to the area most susceptible to recurrence while delivering a lower dose to the normal tissues surrounding the target. Furthermore, BRT enables a short overall treatment time, and the rate of local control is comparatively high. Given the data in the literature, we can conclude that BRT is generally the only radiotherapeutic option available when high grade STS recurs in a previously irradiated area [13–15, 20–22].

BRT usually involves the temporary or permanent surface, intracavitary, or interstitial application of radioisotopes. Many types of BRT are being investigated, including interstitial high dose rate BRT (HBRT), low dose rate brachytherapy (LBRT), intraoperative HBRT, and combinations of these modalities. However, no clinical trial has compared HBRT with LBRT for recurrent STS. In some pediatric cases, HBRT with ^192^Ir has been used either alone or in combination with EBRT in the management of STS and has been shown to provide good local control and overall survival rates. Gustavo et al. [16] reported their experience of treating STS with HBRT alone or in combination with EBRT in 18 pediatric patients (age, 2–16 years; median, 11 years) who had intermediate to high grade tumors at the time that HBRT was performed. With a median follow-up of 79.5 months (range, 12–159 months), overall survival rates at 5 and 10 years were 84.4 and 72.4 % and the overall local control rates for the HBRT group (eight cases) and the HBRT plus EBRT group (10 cases) were 100 and 90 %, respectively. Merchant et al. [10] previously reported that, for pediatric patients with STS, BRT was an excellent treatment option. In their study, 31 patients of median age 11 years (range, 1–21 years) were managed with BRT initially or at the time of recurrence using ^125^I or ^192^Ir in a temporary or permanent manner. At the time of follow-up, 25 patients were alive, with a median survival time of 34 months.

In the present study, all patients were treated with interstitial permanent implantation of ^125^I seeds. Interstitial ^125^I seed implantation is a method of LBRT that for many years has been a gold standard prostate BRT in low risk patients. ^125^I seeds implanted in a tumor continuously emit low dose X-rays and γ-rays; during the half-life of ^125^I, they deliver a dose of 160–180 Gy to the local tissues, sparing adjacent normal structures and medical personnel. This slow emission allows any normal tissue that does receive a sublethal or potentially lethal dose of radiation to repair and recover [23]. Also, continuous low dose irradiation may reduce the oxygen enhancement ratio, which may improve the efficacy of the treatment in hypoxic portions of the tumor [24]. In addition, the therapeutic benefit may theoretically be enhanced by natural increases in local dose after radiation-induced tumor shrinkage brings the ^125^I seeds closer together [25]. Finally, permanent interstitial ^125^I seed implantation is a quick, 1 day therapy with a low complication rate both during and after the procedure [6–9]. Taking all of these benefits into account, permanent ^125^I seed implantation may be well suited for the management of pediatric STS, especially in patients with recurrence and/or metastasis who have already received multimodal treatment.

Hentz and Barrett [26] reviewed eight pediatric patients with rhabdomyosarcoma who were treated with temporary LBRT using ^125^I, with promising results. The local recurrence rate was 12.5 % after treatment, and the side effects were endurable, especially in patients who had no prior history of irradiation. Zhang et al. [27] reported a total survival rate of 88 % in eight children during a median follow-up of 43 months. Li et al. [28] demonstrated that when a combination of ^125^I seeds and an artificial prosthesis was used to replace tumor tissue in three patients, limb function recovered well. The patients remained disease-free for 14–18 months and experienced no severe complications such as infection or wound bleeding.

The success of ^125^I BRT mainly depends on the precise location of the implant needles. In the present study, ^125^I seed implant was guided by CT or ultrasound, which ensures accurate placement of seeds within a known volume of tumor [29, 30]. Optimization of seed distribution and dose homogeneity can easily be achieved by adjusting the position of each needle and seed during implant according to the pretreatment plan. In this respect, ^125^I seed implantation is a safe radiotherapy and delivers more conformal radiation. However, few authors have reported on percutaneous seed implantation as a sole therapy for recurrent or metastatic STS under image guidance, especially in pediatric patients. In the present study, we performed permanent interstitial ^125^I seed implantation as a salvage therapy for pediatric recurrent and metastatic STS. Our results demonstrate satisfactory tumor local control and overall survival rates. Organ function and cosmetic appearance were maintained and uncompromised.

## Conclusions

Image guided permanent interstitial ^125^I seed implantation as a sole salvage modality is a feasible, minimally invasive treatment for pediatric recurrent or metastatic STS, with few complications. It avoids the morbidity associated with further surgery or EBRT and achieves high survival and local control rates with endurable toxicity. The long-term results of this promising procedure with a greater number of cases are needed to reach a definite conclusion.

## Abbreviations

3D-TPS: three-dimensional radiation therapy planning system
AJCC: American Joint Committee on Cancer
BRT: interstitial permanent brachytherapy
CDU: color Doppler ultrasound guided
CI: confidence interval
CR: complete response
CT: computed tomography
CTx: chemotherapy
EBRT: external beam radiotherapy
GTV: gross tumor volume
HBRT: high dose rate BRT
LBRT: low dose rate brachytherapy.
LR: local recurrence
m: months
MM: multiple metastasis
MRI: magnetic resonance imaging
OAR: organs at risk
PD: progressive disease
PR: partial response
RR: response rate
RTOG: Radiation Therapy Oncology Group
SD: stable disease
STSs: soft tissue sarcomas
